# Differentiation of *Candida albicans* complex species isolated from invasive and non-invasive infections using *HWP1* gene size polymorphism

**DOI:** 10.18502/cmm.7.2.7034

**Published:** 2021-06

**Authors:** Kourosh Salehipour, Shima Aboutalebian, Arezoo Charsizadeh, Bahram Ahmadi, Hossein Mirhendi

**Affiliations:** 1 Department of Medical Parasitology and Mycology, School of Medicine, Isfahan University of Medical Sciences, Isfahan, Iran; 2 Immunology, Asthma, and Allergy Research Institute, Tehran University of Medical Sciences, Tehran, Iran; 3 Department of Medical Laboratory Sciences, Faculty of Paramedical, Bushehr University of Medical Sciences, Bushehr, Iran; 4 Core Facilities Research Laboratory, Mycology Reference Laboratory, Isfahan University of Medical Sciences, Isfahan, Iran

**Keywords:** *Candida albicans species complexes*, *Candida africana*, *Candida dubliniensis*, *HWP1* gene

## Abstract

**Background and Purpose:**

Taxonomy of *Candida* is controversial and has changed due to the investigation of the novel species. *Candida africana* and *Candida dubliniensis* are new members of the *C. albicans* complex that are currently gaining both clinical and epidemiologic significance. This study aimed to report the prevalence of *C. africana* among the strains isolated from patients using hyphal wall protein 1 (*HWP1*) gene size polymorphism.

**Materials and Methods:**

In total, 235 yeasts confirmed as *C. albicans* complex based on chromogenic media and internal transcribed spacers sequencing isolated from various clinical forms of invasive and non-invasive candidiasis mainly candidemia were re-identified using *HWP1* gene polymorphisms. The *HWP1*-polymerase chain reaction amplicons were re-confirmed by sequencing and BLAST analysis.

**Results:**

Based on the *HWP1* gene size polymorphism, 223 strains were identified as *C. albicans* (94.89%) from which 7 isolates produced two DNA fragments (850 and 941 bp). The *C. dubliniensis* (n=4, 1.7%), *C. africana* (n=1, 0.42%), and mix of *C. albicans* and *C. africana* (n=7, 2.97%) were also identified.

**Conclusion:**

It can be said that *C. albicans* remains the most common *Candida* species, while *C. dubliniensis* and *C. africana* are rarely found among the patient isolates. Due to limited information on the molecular epidemiology of this novel yeast, more studies using molecular methods are recommended.

## Introduction

Incidence rate of infections caused by various yeasts species has increased considerably in the past decades [ 1 ]. Classification of *Candida albicans* as the most common cause of invasive fungal infections has been subjected to significant changes describing new species, such as *Candida dubliniensis* and *Candida africana*, as the cryptic species complexes [ 2 , 3 ]. According to the limited number of studies performed to date, *C. africana* is reported to have a strong association with human genitals, and it is rarely isolated from other body sites [ 4 ]. Accordingly, *C. dubliniensis* and *C. africana* have received less attention, compared to *C. albicans* and there is a lack of experimental and clinical evidence about their pathogenic potential. 

According to the previous studies, *C. dubliniensis* and *C. africana* are inherently susceptible to azole and polyene antifungal drugs. However, some reports have shown that the antifungal susceptibility patterns of *C. africana* and *C. dubliniensis* are slightly different from those of *C. albicans* [ 5 , 6 ]. Moreover, based on previous studies, some *C. africana* isolates have been classified as resistant to itraconazole, fluconazole, voriconazole, clotrimazole, 5-flucytosine, and Terbinafine [ 4 , 7 , 8 ]. Echinocandins is the first-line antifungal drug for the treatment of *Candida* infections and has shown prolonged post antifungal effect and concentration-dependent killing activity against the majority of *Candida* species, including the *C. albicans* complex [ 9 , 10 ]. 

Phenotypic characteristics do not allow differentiation between the members of closely related *C. albicans* complex species. More reliable tests are based on molecular techniques, such as specific polymerase chain reaction (PCR) amplification of the hyphal wall protein 1 (*HWP1*) gene [ 11 ]. The *HWP1* gene has been proposed as the molecular target for discriminating between *C. albicans* species complex based on its size polymorphism as it has 941/850 base pair (bp) for *C. albicans*, 569 bp for *C. dubliniensis*, and ∼700 bp for *C. africana* [ 11 ].

Although epidemiological and clinical data suggest that *C. africana* has a worldwide distribution, little is known about the frequency of *Candida* isolated from systemic candidiasis in Iran [ 12 ]. Hence, this study was carried out to investigate the microbial epidemiology of *C. albicans* complex species among different clinical specimens, especially those strains isolated from systemic candidiasis.

## Materials and Methods

### Candida albicans complex isolates and strains

The majority of *Candida* samples had already been isolated from the patients with systemic candidiasis admitted to the neonatal and pediatric ICUs of Children’s Medical Centre, Tehran, Iran, and identified as *C. albicans* mostly by internal transcribed spacers sequencing and/or matrix-assisted laser desorption ionization-time of flight [ 13 ]. In addition, a part of the samples was isolated from vulvovaginal candidiasis and candiduria from the patients in Al-Zahra Hospital, Isfahan, Iran. The *C. albicans* (ATCC 64553), *C. dubliniensis* (ATCC 2018), and two isolates of *C. africana* (GenBank accession number: MG434677 and MG434680) were used as the positive controls.

### Molecular identification

The colonies conserved at -20 ºC freezer were subcultured on CHROMagar *Candida* [ 15 ], and DNA was extracted from a single colony by boiling method [ 14 ]. A fragment of the *HWP1* gene was amplified using CR-f (5'- GCT ACC ACT TCA GAA TCA TCATC-3') and CR-r (5'- GCA CCT TCA GTC GTA GAG ACG-3') primers [ 11 ] in the following thermal conditions: 5 min at 95 ºC, followed by 35 cycles of 40 s at 94 ºC, 45 s at 60 ºC, and 60 s at 72 ºC as well as a final extension of 5 min at 72 ºC. The reaction mixture contained 7.5 μL of 2 master mix (Ampliqon, Denmark), 0.33 μM of each primer, and 2 μl of DNA in a total volume of 15 μl. It should be mentioned that appropriate positive and negative controls were used for each PCR run. 

An aliquot of 5 μl of each sample was added to 1.5% agarose gel containing 0.5 μg/ml of ethidium bromide. It was electrophoresed for "2 h in 100 V" to"90 min in 120 V" and visualized under UV light documentation. Species identification was performed based on the size polymorphism of the *HWP1* gene in different species, i.e. *C. albicans* (∼940/850 bp), *C. dubliniensis* (∼570 bp), and *C. africana* (∼700 bp) [ 11 , 16 ]. The *HWP1*-PCR product identified as *C. africana* was subjected to sequencing with the above-mentioned forward primer and the result was analyzed by Basic Local Alignment Search Tool (http://blast.ncbi.nlm.nih.gov/Blast).

## Results

In this study, a total of 235 *Candida albicans* isolates were re-identified based on *HWP1* gene polymorphisms. The isolates were collected from patients with systemic candidiasis (n=150), vulvovaginal candidiasis (n=60), and candiduria (n=25). The age of patients with candidiasis ranged from 1 to 78 years and the majority of them were female (n=154, 65.53 %).

**Figure 1:**
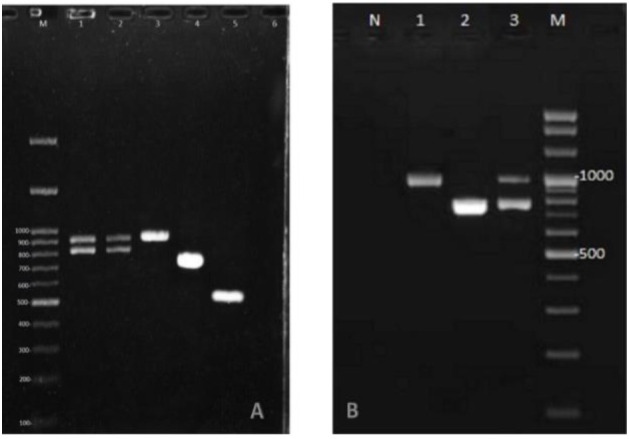
Electrophoretic profile of hyphal wall protein 1 amplification in some clinical isolates. A) Lane M: 100 bp molecular size marker, lanes 1 and 2: heterozygous isolates of *Candida albicans* (∼850 and 940 bp), lane 3: homozygous isolate of *C. albicans* (∼940 bp), lane 4: *Candida africana* (∼700 bp), lane 5: *Candida dubliniensis* (∼570 bp), and lane 6: negative control. B) Lane 1: negative control, lane 2: homozygous isolate of *C. albicans* (∼940 bp), lane 3: *C. africana* (∼700 bp), *C. albicans*, and *C. africana* (dual bands), and lane M: 100 bp molecular size marker." to "Lane N: negative control, lane 1: homozygous isolate of *C. albicans* (∼940 bp), lane 2: *C. africana* (∼700 bp), lane 3: *C. albicans* and *C. africana* (dual bands), and lane M: 100 bp molecular size marker.

Based on *HWP1* gene amplification, the species distribution was as follows: *C. albicans* (n=223, 94.89%), from which 7 isolates produced two different DNA fragments (850 and 941 bp), *C. dubliniensis* (n=4, 1.7%), *C. africana* (n=1, 0.42%), and the mix of *C. albicans* and *C. africana* (n=7, 2.97%) (Figure 1). The amplicon of the single pure *C. africana* isolate was subjected to PCR-sequencing. The obtained sequences showed 99.71% identity with an isolate of *C. africana* (MN817936.1) with an E-value of 99.42 and 100% coverage and the sequence was inserted in GenBank under accession numbers MZ578437. It is noteworthy that BLAST analysis of the obtained sequence confirmed the identity. 

This *C. africana* was obtained from the urine specimen of a 45-year-old female with diabetes. All isolates of *C. dubliniensis* and all samples with a mix of *C. albicans* and *C. africana* were collected from the patients with systemic candidiasis. The results of the molecular analysis are shown in Table 1.

**Table 1:** Distribution of *Candida albicans* species complexes in this study

	*Candida albicans*	*Candida dubliniensis*	*Candida africana*	Mix of *C. albicans* and *C. africana*
Systemic candidiasis	139	4	-	7
Candiduria	24	-	1	-
Vulvovaginal candidiasis	60	-	-	-
Total	223	4	1	7	

## Discussion

The incidence of mild to severe fungal infections has dramatically increased worldwide in the last several decades. Fungal species distribution varies owing to the hospital, hospitalization unit, and geographical area [ 17 ]. Invasive candidiasis is a considerable cause of morbidity and mortality, especially amongst patients suffering from immunodeficiency [ 18 ]. The *C. albicans* complex is one of the major fungal groups, which is involved in more than 50% of *Candida* infections, pointing out their significant prevalence among human beings [ 19 ]. 

The differences in adherence ability, pathogenicity, and biofilm formation observed between *C. albicans* and *C. africana* highlight the necessity of discriminating them in clinical laboratories [ 20 ]. Therefore, this study aimed to identify the archived cryptic specimens belonging to the *C. albicans* complex isolated from clinical samples of hospitalized patients to demonstrate the existence of species that are not routinely identified and reported.

The *HWP1* is a particular target for differentiation of the *C. albicans* complex species i.e. *C. albicans*, *C. dubliniensis*, and *C. africana* [ 7 ]. In the present study, *C. albicans* (94.89%) was the predominant species among the 235 isolates, which is in line with the results of some previous studies [ 12 , 16 , 21 - 24 ]. In the present research, the majority (97.3 %) of *C. albicans* with 941 bp DNA fragments were homozygous, while 2.97% (n=7) of them produced two DNA fragments of 850 and 941 bp, demonstrating heterozygosity at *HWP1* locus. The 850 bp DNA fragment is considered a novel allele of the *HWP1* gene [ 22 ]. 

Nouraei et al. [ 25 ] evaluated the exoenzyme activity of 60 *C. albicans* species consisting of 30 homozygous and 30 heterozygous strains. They found that the homozygous strains of *C. albicans* had more phospholipase and proteinase exoenzyme activity than heterozygous strains in different ranges, while no signiﬁcant statistical differences were observed between the strains in terms of virulence factors. Further studies are needed to clarify the probabilistic pathogenic role of these homozygous or heterozygous strains.

In this study, *C. africana* (3.4%) had a higher prevalence rate than *C. dubliniensis* (1.7%). This result corroborates those of the previous research performed in Iran [ 7 , 12 , 26 - 28 ]. However, the higher isolation rate of *C. dubliniensis* over *C. africana* has also been reported in other studies [ 21 , 29 , 30 ]. In a study conducted by Romeo et al. [ 31 ], the frequency of *C. africana* (7.2%) was higher than that of *C. dubliniensis* (2.9%) among the *Candida* strains isolated from 498 clinical specimens collected from various patient groups [ 31 ]. The *C. dubliniensis* is less prevalent than *C. albicans* and shows phenotypic similarities with *C. albicans*, which may invade sterile body sites, such as mucosal surfaces, blood, central nervous system, and pleural fluid, with mortality rates similar to *C. albicans* [ 32 - 35 ].

Although *C. africana* has a worldwide distribution, an epidemiological meta-analysis showed that its overall prevalence rates in Iran and Honduras were higher, compared to other countries worldwide [ 5 ]. Shokoohi et al. [ 26 ] reported that one of the largest clusters of *C. africana* isolates was from Iran with a prevalence rate similar to those reported from some other countries indicating that this yeast may be more locally or regionally prevalent [ 26 ]

Hana et al. [ 21 ] reviewed the global epidemiological status of *C. africana* reported between 2010 and 2019 from more than 11 different countries (Senegal, Nigeria, Cameroon, Algeria, United Kingdom, Argentine, Colombia, USA, Iran, China, and Turkey). They found that the majority of *C. africana* strains were identified in America (35/90-38.8%), followed by Asia (27/90-30%), Europe (15/90-16.6%), and Africa (13/90-14.4%). Despite its worldwide distribution, the majority of *C. africana* isolates have been isolated from vulvovaginal candidiasis (60/90-66.6%) followed by nosocomial origins (11/90), balanoposthitis (5/90), blood (1/90), cerebral liquid (1/90), buccal (1/90), and urine (1/90) [ 21 ].

Based on the results of global epidemiological studies, most of the *C. africana* strains have been isolated from vulvovaginal specimens [ 36 ]. However, in agreement with the study conducted by Yazdanpanah et al. [ 37 ] and Gumral et al. [ 38 ], the results of our assay revealed that no *C. africana* was recovered from the vaginal specimens. The distribution of *C. africana* may be partially based on geographical variation, although a larger number of vulvovaginal samples are needed to confirm this hypothesis. We also identified *C. africana* among the patients with candiduria, suggesting that this fungus can also be associated with a wider clinical spectrum [ 31 ].

In this investigation, molecular identification demonstrated seven co-infections by *C. africana* and *C. albicans* in patients with systemic candidiasis. An attempt to discriminate species in mixed infection/colonization, especially in children, is important for clinicians as they could differ both in virulence and spectrum of antifungal. Consequently, the lack of specific microbiological data could force physicians to empirically treat life-threatening mycoses with broad-spectrum antifungal medications, which would impact the existing issues with antifungal resistance.

## Conclusion

While *HWP1* size polymorphisms are a simple and cost-effective method for the differentiation of *C. africana* and *C. dubliniensis* from *C. albicans*, *C. africana* was detected in 3.4% of the isolates. This means that this species is not uncommon in Iranian patients.

## Acknowledgement

This study was financially supported by Isfahan University of Medical Sciences, (IUMS), Isfahan, Iran. Ethical approval of the study was obtained from the Ethics Committee of IUMS (IR.MUI.RESEARCH.REC.1398.728(.

## Authors’ contribution

H.M. designed the study. A.C. and B.A. provided the isolates. S.A. and K.S. performed the experiments. S.A. and H.M. prepared the draft of the paper. All authors assisted in the edition and revision of the manuscript.

## Conflict of Interest

All authors report no potential conflicts of interest. The authors alone are responsible for the content and writing of the paper.

## Financial disclosure

The authors received no external funding for this study.
